# Perception of the compatibility of Quebec residency program characteristics with the advanced access model: a cross-sectional study

**DOI:** 10.1186/s12875-024-02386-5

**Published:** 2024-05-10

**Authors:** Marie-Ève Boulais, Nadia Deville-Stoetzel, François Racine-Hemmings, David Perrier, Élisabeth Martin, Étienne Boyer-Richard, Raffaele Di Zazzo, Eve Labbe, Mylaine Breton, Isabelle Gaboury

**Affiliations:** 1 Department of Family Medicine and Emergency Medicine, Université de Sherbrooke, Quebec, Canada; 2 Centre de recherche du Centre Hospitalier Universitaire de Sherbrooke (CRCHUS), Quebec, Canada; 3 Department of Community Medicine, Université de Sherbrooke, Quebec, Canada

**Keywords:** Advanced access, Primary care, Medical education, Cross-sectional survey, Interprofessional collaboration, Care management

## Abstract

**Background:**

The advanced access (AA) model is among the most recommended innovations for improving timely access in primary care (PC). AA is based on core pillars such as comprehensive planning for care needs and supply, regularly adjusting supply to demand, optimizing appointment systems, and interprofessional collaborative practices. Exposure of family medicine residents to AA within university-affiliated family medicine groups (U-FMGs) is a promising strategy to widen its dissemination and improve access. Using four AA pillars as a conceptual model, this study aimed to determine the theoretical compatibility of Quebec’s university-affiliated clinics’ residency programs with the key principles of AA.

**Methods:**

A cross-sectional online survey was sent to the chief resident and academic director at each participating clinic. An overall response rate of 96% (44/46 U-FMGs) was obtained.

**Results:**

No local residency program was deemed compatible with all four considered pillars. On planning for needs and supply, only one quarter of the programs were compatible with the principles of AA, owing to residents in out-of-clinic rotations often being unavailable for extended periods. On regularly adjusting supply to demand, 54% of the programs were compatible. Most (82%) programs’ appointment systems were not very compatible with the AA principles, mostly because the proportion of the schedule reserved for urgent appointments was insufficient. Interprofessional collaboration opportunities in the first year of residency allowed 60% of the programs to be compatible with this pillar.

**Conclusions:**

Our study highlights the heterogeneity among local residency programs with respect to their theoretical compatibility with the key principles of AA. Future research to empirically test the hypotheses raised by this study is warranted.

**Supplementary Information:**

The online version contains supplementary material available at 10.1186/s12875-024-02386-5.

## Introduction

Advanced access (AA) is among the most recommended innovations for improving timely access in primary care (PC) [1–3]. Timely access, defined as patients being able to access care when they need professional attention, is a core attribute of the patient-centered medical home model [4, 5]. The AA model ensures patients obtain an appointment within an appropriate period of time with the right professional for their condition who responds in an optimal way to their expected needs [2, 6, 7].

The AA model was developed based on five pillars: (1) balancing appointment supply and demand; (2) reducing the backlog of previously scheduled appointments and setting up a communication plan; (3) reviewing the appointment scheduling system; (4) integrating interprofessional practices; and (5) developing contingency plans [2]. Each pillar is operationalized through a series of principles. Since the model’s development over 20 years ago, PC clinics have evolved towards interprofessional practice models and now rely on contemporary electronic solutions to manage and deliver care. In light of this, the AA model has recently been revisited [7]. Table 1 presents a summary of the five revised pillars and examples of how their related principles are operationalized in practice.

**Table 1:** Five revised pillars of the advanced access (AA) model

Pillar	Description^a^	Examples of operationalization
1 – Comprehensive planning for needs, supply and recurring variations	The clinical team plans appointment availability according to patient needs and characteristics, taking into account seasonal fluctuations in supply and demand.	Extended visit intervals are used to decrease demand for visits. Doctor’s scheduling systems are redesigned to increase supply.
2 – Regular adjustment of supply to demand	Service availability is regularly updated by the clinical team to correspond with patients’ needs.	Add resources or increase the supply of visits for a limited period of time.
3 – Processes of appointment booking and scheduling	The appointment scheduling process enables patient communication with the clinic and ensures clinical team members are available according to patient needs.	Plan physicians’ schedules over 2–4 weeks. Smooth out the demand for visits in order to offer same-day appointments for acute and urgent cases.
4 – Integration and optimization of collaborative practice	Interprofessional collaboration is established in the clinic to ensure health care and services are provided based on patient needs and the characteristics of team members (i.e. roles, responsibilities, skills).	Implement collective prescriptions with nurses. Ensure that the role of each professional is well-known and understood.
5 – Communication about AA and its functionalities	Patients and clinical team members are given information on AA principles and practice and updated on changes within the organisation.	Provide educational material to inform patients about AA. Measure patient satisfaction and care experiences with respect to AA.

^a^ Descriptions are based on definitions of pillars redefined by Breton et al. [6].

Family medicine groups (FMGs) are the principal model for publicly-funded interdisciplinary primary healthcare clinics in Quebec [8]. Within this model, physicians and the Quebec Ministry of Health and Social Services (MHSS) are bound by a contractual relationship. Physicians are responsible for delivering high quality, continuous and accessible PC to a given patient group, in exchange for which the MHSS provides funding for support staff and allied healthcare professionals within the clinic. Quebec family medicine residency programs are centered around university-affiliated family medicine groups (U-FMGs). U-FMGs are FMGs with a triple mission: providing PC, teaching PC to physicians in training as well as other health professionals and participating in the development and application of knowledge through research [9]. U-FMGs are exemplary organizations that expose trainees to best practices during their training programs. U-FMGs adhere to the principles and vision of the medical home [10], among which is accessibility of care. Some scholars have hypothesized that exposing future physicians to AA during their training promotes its implementation within health systems. Learning to provide timely care, as advocated by AA, is a goal of postgraduate family medicine training but is not a clearly stated goal of undergraduate medical programs [11]. Given that residents have little to no exposure to AA practices prior to entering residency, actively teaching the model is necessary and has been identified as a promising way to ensure its continuation when residents enter practice [12]. Some scholars hypothesize that this would contribute to residents’ sense of efficacy and responsibility, better preparing them for future practice. Therefore, residency represents an opportunity for outreach of AA as a best practice [12, 13].

Furthermore, it is expected that patients under resident care would benefit from accessible and continuous care at the same level as has been demonstrated by physicians in practice [14–21]. However, the academic realities of Canadian family medicine residency programs potentially conflict with the principles of AA. For example, residents typically see fewer patients each day than staff. However, their capacity grows during the residency program. Residents also have limited availability over time, often spending weeks to months away from their clinics due to other training requirements. This often depends on whether the residency curriculum is horizontal, vertical or mixed. A horizontal curriculum involves frequent (minimally weekly) variations in exposure to various settings and domains of care on a longitudinal basis. Thus, a resident in a horizontal curriculum would normally offer availability for their patients on a weekly basis. In contrast, a vertical curriculum concentrates exposure in one care domain and context over a period of a few weeks (typically 4 to 12), which strictly limits the capacity of a resident to see patients on a timely basis. Finally, the success of AA relies heavily on interprofessional collaboration and the ability of physicians to delegate and share care with other professionals. However, the context of residency practice is dictated by the learning needs of trainees. Thus, residents must practice certain tasks that are typically delegated or shared with other professionals within the clinic (e.g. monitoring and adjusting medication for diabetic patients), thereby limiting opportunities for collaboration, particularly at the beginning of the residency program.

Although the impact of AA on PC practice is a burgeoning field of research, less is known about the successes and failures of implementing AA among local residency programs. Only a small number of studies have shown that the principles of AA can be successfully integrated into a residency program and schedule [15, 22–24]. AA implementation allowed for decreased waiting times and increased continuity despite the part-time presence of residents [15, 25]. Two other studies [23, 24] also confirmed increased continuity as well as reduced time to the third next available appointment (a common marker of AA success) [21]. Notably, patient satisfaction was unchanged in these studies. These studies commonly noted small sample sizes, irregular resident availability and organizational constraints as limitations to both AA implementation and interpretation of study results.

The cross-sectional nature of previous studies does not allow for a comprehensive evaluation of the impact of implementing the principles of AA within a residency schedule. To our knowledge, no study has been able to assess the variations between different local residency programs or the applicability of the AA principles to a PC residency practice. The academic context of residency programs described above led us to question whether the factors facilitating the implementation of AA among staff can be applied directly to the residency context [26, 27]. Such an analysis would help us better understand the nature of facilitators and constraints as well as their impact on residents’ AA practices. The objective of this study is to determine the theoretical compatibility of Quebec residency programs with the key principles of AA.

## Methods

### Design and setting

A cross-sectional study was conducted based on an open e-survey hosted on a web platform (Survey Monkey) and distributed between December 2020 and April 2021. All chief residents and academic directors working at 46 U-FMGs across the province of Quebec were invited by the research team via email to complete the anonymous online questionnaire on a voluntary basis.

The de novo questionnaire was developed by the research team, which included clinicians, AA experts, a local residency program expert and four family medicine residents. Content validity was evaluated qualitatively by the research team committee, which included experts on AA, interprofessional collaboration and survey reporting (*n* = 3), physicians (*n* = 2) and family medicine residents (*n* = 7) [28]. The questionnaire (Appendix 1) took approximately 15 min to complete and included 32 questions that aimed to describe the local residency program through each AA pillar and their related principles, including resident appointment supply and appointment length during the residency journey, resident panel size, program organization (vertical, horizontal or mixed), schedule availability (proportion left open for semi-urgent or urgent patient needs, proportion of time slots dedicated to residents’ own patients, number of weeks open for appointments) and opportunities to collaborate with other professionals, such as nurses, social workers and pharmacists, as these are members of basic interprofessional PC teams in the province. The fifth pillar, communication about AA, was evaluated based on the training received on AA. A unique user identifier was assigned to each respondent. The CHERRIES checklist was considered for reporting the survey results [29].

### Analysis

All variables were described as frequencies and proportions using descriptive statistics. The surveyed characteristics of the local residency programs were grouped under the associated AA pillar. A compatibility algorithm was developed by expert consensus (MEB, FRH, NDS, MB and IG) using an iterative reflexive approach [30]. A three-level score (compatible, moderately compatible or not very compatible with the pillars of AA) was assigned to each group of characteristics under each pillar (Table 2).

**Table 2:** Algorithm of compatibility of local residency programs

Pillar	Compatibility level with AA	Factors considered
1. **Planning for needs, supply and recurring variations**	Compatible	1) at least two appointment slots per patient assigned to a resident per year AND 2) a horizontal rotation sequence
	Moderately compatible	1) at least two appointment slots per patient AND a mixed rotation sequence OR 2) fewer than two appointment slots per patient AND a horizontal rotation sequence
	Not compatible	Any other combination of numbers of available appointment slots and rotation sequences
2. **Regular adjustment of supply to demand**	Compatible	Maximum of 7 days between two clinics during a rotation. One clinic per week to increase patient continuity is the standard for U-FMGs [31, 32]
	Moderately compatible	N/A
	Not compatible	More than 7 days between two residency clinics
3. **Processes of appointment booking and scheduling**	Compatible	1) at most 2 weeks open in advance for scheduling appointments AND 2) over 20% of appointments kept available 48 h in advance for emerging needs AND 3) over 75% of appointments dedicated to patients assigned to a resident
	Moderately compatible	1) at most 4 weeks open in advance for scheduling appointments AND 2) less than 20% of appointments kept available 48 h in advance for emerging needs OR 3) less than 75% of appointments dedicated to patients assigned to a resident
	Not compatible	Any other combination of factors with respect to opening appointments and limiting appointments to affiliated patients
4. **Integration and optimization of collaborative practice**	Compatible	1) possibility of engaging in joint patient care with a non-physician professional at the beginning of the first residency year AND 2) joint patient care with every professional present in the clinic
	Moderately compatible	1) possibility of engaging in joint patient care at the beginning of the first residency year but not with every professional present in the clinic OR 2) joint patient care with all professionals but only during the second residency year or with other restrictions
	Not compatible	Any other combination of joint patient care

## Results

Of the 46 U-FMGs, 44 responded to the e-survey, for an overall response rate of 96%. Sufficient responses were obtained from all responding clinics and were used for the analysis. Selected local residency program characteristics are summarised in Table 3.

**Table 3:** Local residency program characteristics

Number of residents per U-FMG (Number of U-FMGs)	≤ 10 (8) 11–20 (14) 21–30 (16) 31–40 (4) 41 < (2)	
	**median (min**–**max)**	
Number of residents per year in the model	PGY^a^ 1	10 (3–26)
	PGY 2	10 (0–28)
Number of patients per resident	PGY 1	100 (50–200)
	PGY 2	125 (60–200)
Number of half-days in clinic per month during internship in U-FMG	12 (3–28)	
Number of half-days in clinic per month during non-U-FMG internship	4 (0–12)	
Number of months in U-FMG internship during 2-year residency	12 (5–20)	
Number of months in non-U-FMG internship during 2-year residency	9 (5–21)	
Proportion of schedule left open for urgent or semi-urgent patient needs	15 (0–50)	
Number of weeks open for appointments	4 (2–12)	
Proportion of resident time slots dedicated to their own patients	60 (15–100)	

^a^ PGY, postgraduate year

Overall, no U-FMGs were compatible with all four pillars first considered based on the compatibility algorithm. Of the 44 clinics evaluated, three clinics (7.5% of surveyed clinics) showed compatibility with three of four pillars; 15 clinics (37.5%) with two of four pillars; 13 clinics (32%) with one pillar and nine clinics (22.5%) with no pillars. Table 4 presents the results for each pillar and principle considered for the compatibility analysis.

**Table 4:** Results and status of advanced access compatibility for each pillar, n (%)

Pillar	n (%)	Status
**1) Comprehensive planning for needs, supply and recurring variations**		
Model of local residency program offered in the U-FMG		Compatible: 9 (20) Moderately compatible: 14 (32) Not very compatible: 21 (48)
Horizontal	13 (30)	
Vertical	20 (45)	
Mixed	11 (25)	
Local residency program allows for two or more slots on average per patient assigned		
PGY^a^ 1	20 (67)	
PGY 2	24 (89)	
**2) Regular adjustment of supply to demand**		
Number of days between two residency shifts in clinic		
≤ 7	21 (55)	Compatible: 21 (55)
More than 8	17 (45)	Not very compatible: 17 (45)
**3) Processes of appointment booking and scheduling**		
Number of weeks open for appointments		Compatible: 7 (18) Moderately compatible: 14 (35) Not very compatible: 19 (47)
2	10 (25)	
3–4	24 (60)	
More than 4	6 (15)	
Proportion of schedule left open for urgent or semi-urgent patient needs		
More than 20%	9 (25)	
10–19%	16 (44)	
0–9%	11 (31)	
Proportion of time slots dedicated to patients assigned to a resident		
More than 75%	16 (42)	
50–74%	11 (29)	
Less than 50%	11 (29)	
**4) Integration and optimization of collaborative practice**		
Possibility for joint follow-up with another professional other than a physician		Compatible: 16 (40) Moderately compatible: 19 (47) Not very compatible: 5 (13)
Beginning of PGY 1	25 (62)	
End of PGY 1	2 (5)	
During PGY 2	2 (5)	
On a patient basis	11 (28)	
Possibility for joint follow-up with		
• Clinician nurse	39 (100)	
• Auxiliary nurse	35 (87)	
• Social worker	38 (95)	
• Pharmacist	37 (92)	
• Nurse practitioner	27 (67)	
• Nutritionist	20 (51)	
• Physiotherapist•	7 (18)	
• Occupational therapist	1 (3)	
• Psychologist	22 (56)	
**5) Communication about advanced access and its functionalities**		
Training on advanced access provided	23 (52)	Compatible: 18 (41) Moderately compatible: 5 (11) Not very compatible: 21 (48)
Compulsory training on advanced access	18 (41)	

^a^ PGY, postgraduate year

For the first pillar, comprehensive planning for needs, supply and recurring variations, only one quarter of programs were compatible, mostly because of the predominance of vertical and mixed models among the surveyed programs (70% of clinics). For the second pillar, regular adjustment of supply and demand, over half of the programs were compatible as a result of maintaining fewer than 7 days between clinic shifts. For the third pillar, processes of appointment booking and scheduling compatibility, 82% of the programs’ appointment systems were only moderately compatible with the AA model, mainly because three quarters of the programs had an insufficient (less than 20%) proportion of the schedule left open for urgent or semi-urgent appointments. However, a larger proportion (85%) of the programs kept fewer than 4 weeks open for appointments, with one quarter opening their schedules only 2 weeks in advance. The fourth pillar, integration and optimization of collaborative practices, showed the greatest compatibility, with nearly half of the programs being compatible and 60% of the clinics providing opportunities for collaboration starting in the first year of residency. Furthermore, 24 programs (60%) allowed residents to engage in interprofessional follow-ups.

Post-hoc exploratory crosstab analyses were performed to identify relationships between compatibility status for each pillar and program characteristics not included in the program compatibility categorization (number of residents [correlated with clinic size] and AA teaching). No statistically significant associations emerged.

## Discussion

This study is one of the first to map the theoretical compatibility of local residency programs with the pillars of AA. All U-FMGs in the sample had at least moderately compatible characteristics with a minimum of one AA pillar, but none were fully compatible with all pillars. The pillar processes of appointment booking and scheduling most frequently needed improvement, closely followed by planning of supply and demand. According to our data, the way in which patients are assigned to residents and availability planning according to those patients’ needs represent major challenges to the optimal implementation of AA principles by residents.

Regarding planning for need, supply and recurring variations, determining the optimal number of patients assigned to a resident (panel size) to maintain an adequate balance between supply (appointments offered) and demand (patient needs) remains challenging [13]. The need to consider the individual progression of residents and their educational needs further heightens these challenges. Recommendations for established physicians in terms of optimal panel size vary widely [33], but we identified no specific research papers focused on optimal resident panel size. The Fédération des Médecins Omnipraticiens du Québec recommends estimating yearly appointment needs according to patient sociodemographic and medical characteristics (two visits for patients aged 0–5 years, one visit for those aged 6–69 years, three visits for patients with chronic conditions or those over 70 years) [34]. Based on this recommendation and accounting for the proportion of vulnerable patients a resident normally follows during the residency program [35], their limited access to an interdisciplinary team and the need for more frequent follow-ups with trainees for pedagogical purposes, we considered a minimum of two appointments per patient per year to be sufficient. Further research should examine the impact of this cut-off on various access indicators.

In our study, the compatibility of the pillar planning for needs, supply and recurring variations was largely influenced by the type of local residency program. Given its impact on continuity, it is recognized that the horizontal residency model has greater educational scope [36]. However, no study describes its direct impact on access from a patient perspective. We hypothesized that continuous presence in the clinic is more likely to adequately address the key principles of this pillar and could impact other pillars as well. Indeed, the three U-FMGs in the sample with the most highly compatible ratings (3/4) had a horizontal or mixed curriculum. This suggests that a horizontal or mixed curriculum may be a key driver in the implementation of AA principles. However, this remains to be empirically validated.

In terms of ongoing adjustments, we chose to measure only the time between residents’ clinics because of the difficulties in allowing residents sufficient flexibility to regularly modify their schedule according to demand. Program management constraints, such as advance internship rotation planning, advance pairing of residents with available supervising staff and required exposure to areas of care outside of the U-FMG, make residents’ schedules inherently less flexible than those of staff. The perceived immutability of these constraints led us to believe that there were few opportunities to optimize practices for this pillar. Thus, reducing the planned delay between available appointments was the only option considered. An alternative, but more complex, approach would be to promote flexible supervisory partnerships wherein supervisors commit in advance to be available during given time periods when residents may choose to schedule additional clinics in response to increased patient demands.

For the third pillar, processes of appointment booking and scheduling, almost half of the responding clinics were not compatible due to an insufficient proportion of slots reserved for urgent appointments. Theoretically, this pillar should not be directly affected by residency limitations, as reserving appointments could be done regardless of whether residents are continuously available. However, this trend is mirrored in the practice of Quebec family physicians, who overall reserve only 10% of appointments for urgent and semi-urgent patient needs [37]. Therefore, the same barriers to implementation seem to be reflected in residency practices despite theoretical feasibility.

The fourth pillar, integration and optimization of collaborative practice was found to be mostly compatible overall. However, the proportion of compatible programs was lower than expected given the importance placed on collaboration, as evidenced by CanMEDS [38], the repository of competencies expected of a physician. Although CanMEDs deems the collaborator role to be important, practical limitations may exist due to training requirements and organizational constraints. For example, the choice of some U-FMGs to impose limits on collaboration may arise from the need for residents to develop certain skills prior to their delegation as well as administrative and organizational constraints that go beyond those of AA [39]. However, the benefits of collaboration in AA models must be stated; they allow for direct access to the proper professional (e.g. certain problems might be addressed directly by a nurse) and facilitate identification of patients who must see their doctor (serving a triage function) [40]. Given these benefits, along with the educational goals of interprofessional collaboration as described in CanMEDS, we believe it is worthwhile to fully integrate collaboration into medical residency training.

Finally, regarding communication about AA, the rate of U-FMGs teaching AA to residents was rather low (50%), given that several accessible training courses are available to doctors and residents. It is possible that the perceived constraints of applying the AA model in U-FMG practices limit the teaching of AA to residents. Busy residency curricula may also contribute to this issue. However, as many programs currently include formal education sessions on AA early in residency training, this problem seems far from insurmountable. Formal AA teaching in clinics where AA is implemented with residents and staff could be interpreted as a form of explicit role modeling, which is known to enhance learning in clinical settings [41]. This could be expanded on by having senior residents analyze their own accessibility and continuity rates among their patients.

A recurrent theme in our analysis was the often competing objectives of role-modeling the provision of highly accessible care to patients through AA during residency and delivering exemplary postgraduate family medicine training. Residents in U-FMG practices are important PC service providers, but the learning program requirements for safe supervision and varied clinical exposure impose limits to the application of an integral AA model. These limits are described in detail above for each AA pillar.

## Limitations

This study faces limitations that might impact its conclusions and the potential for replication. While the data collection method achieved a commendable response rate, the study is confined by the relatively small number of U-FMGs in the province, totaling 46. Additionally, residency models vary across different jurisdictions, potentially limiting the generalizability of the findings to healthcare systems resembling Canada’s. Furthermore, the exploratory nature of the study, characterized by the lack of established psychometric properties for the questionnaire and the inductive approach to data analysis, represents another limitation. The study, however, has allow us to contrast local residency programs with the principles of AA and learn more about resident training about AA. A critical approach allowed us to identify a series of hypotheses that will be further tested in the second phase of the project. Also, since this survey was conducted in U-FMGs in Quebec, the results may not be applicable to the practices of other local family medicine residency programs in Canada or elsewhere AA is well implemented. Finally, self-reported survey data has some limitations. Such data are susceptible to social desirability bias, memory response bias and possible ambiguities in question interpretation.

## Conclusion

Our study highlights the heterogeneity of local residency programs in Canada’s second most populous province with respect to their compatibility with the pillars of AA. It has allowed us to hypothesize which residency model characteristics better promote access and continuity of care from a patient perspective. Verifying those hypotheses by correlating actual access indicators with local residency program characteristics is warranted. This study allows us to make useful observations on the status of the implementation of AA pillars within local residency programs and the organizational constraints in place and identify the pillars where optimization efforts must be directed.

### Electronic supplementary material

Below is the link to the electronic supplementary material.

Supplementary Material 1

## Data Availability

Not applicable.

## Abbreviations

AA: advanced access
FMG: family medicine group
MHSS: Ministry of Health and Services
PC: primary care
U-FMG: university-affiliated family medicine group
