# Supplementary material for: Perception of the compatibility of Quebec residency program characteristics with the advanced access model: a cross-sectional study
Source: BMC Prim Care. 2024 May 10;25:160. doi: 10.1186/s12875-024-02386-5 (PMC11084022; doi:10.1186/s12875-024-02386-5)
Supplement: Supplementary file 1 — Supplementary Material 1 [file 12875_2024_2386_MOESM1_ESM.docx]

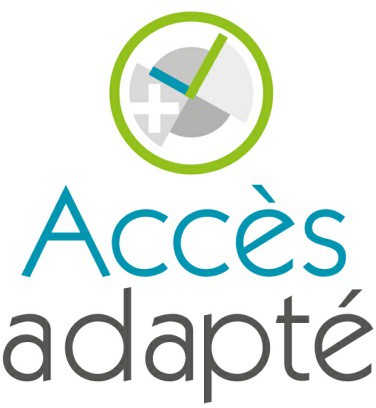


# Access adapted for GMF-U residents.

## GMF-U description

- Which **GMF-U** do you belong to?


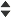


#### What is your **role** in the GMF-U?


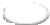
 Medical director


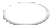
 Resident assistant/resident coordinator


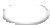
 Other (please specify):

**How many** residents does your FMG-U currently have?

R1:

R2:

**Is** a **resident assigned** to a speciﬁc group of supervisors during his residency?

- Yes, to a **group of** speciﬁc supervisors


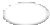
 No, to **a single** supervisor


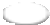
 No, supervision can be carried out by **any supervisor**.

## Residency model for your GMF-U

Which **residency model** (P1-P13) best applies to your GMF-U, depending on the type of internship?

### Choose the most appropriate option.


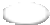
 **Horizontal**: Internship environments that change within the same week or from one week to the next, such as a physician's practice schedule (e.g., 3 days in the office followed by a week of hospital on-call followed by 3 days in the emergency room, etc.).


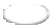
 **Vertical**: Block of consecutive weeks dedicated to one internship environment (e.g.: 4 weeks in the emergency room followed by 4 weeks in the office, etc.).


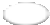
 **Mixed**


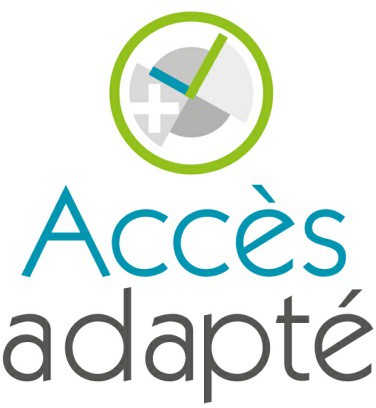


# Access adapted for GMF-U residents.

## Residence model in your GMF-U

#### For the mixed model, **how many periods** of each category do you have? (A period corresponds to a block of 4 weeks, which is used as a time scale in the residence)

Horizontal

Vertical


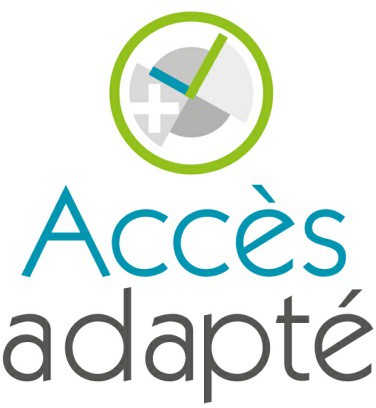


**Access adapted for GMF-U residents.**

Residence model in your GMF-U - continued

How many GMF-U office **half-days** do residents spend in a month in GMF-U (excluding walk-in and specialized clinics)?

Number of half-days

What is the minimum number of office half-days that residents spend in a non-GMF-U month?

Number of half-days

How many months do residents spend on internships at GMF-U?

How many months do residents spend on internships outside GMF-U?

Is there a limit to the number of days I can take off during GMF-U office weeks?


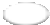
 No
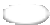
 Yes


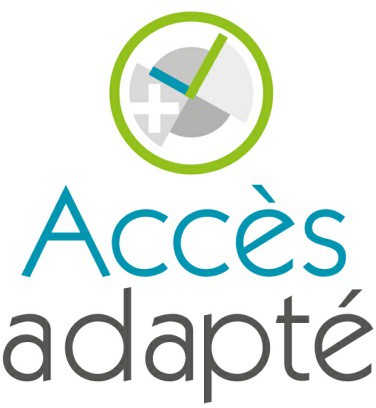


# Access adapted for GMF-U residents.

#### What's the limit?

Maximum number of days of absence in a year


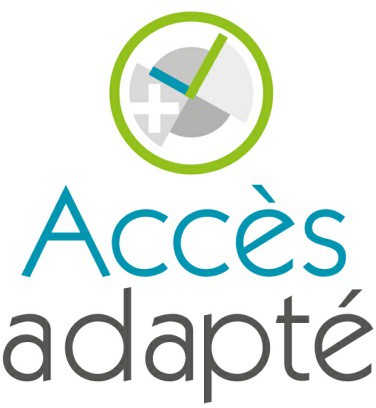


**Access adapted for GMF-U residents.**

Excluding absences for personal reasons, what is the maximum length of time between two residences during a training period **at your GMF-U**?

Maximum duration in days


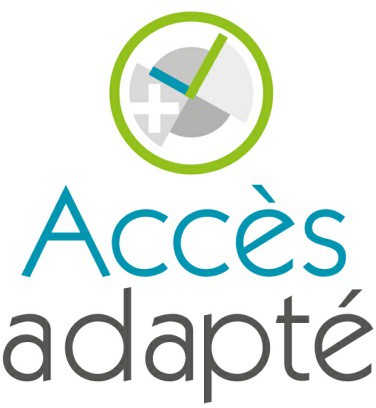


**Access adapted for GMF-U residents.**

Excluding absences for personal reasons, what is the maximum length of time between two residency offices in your GMF-U during an internship **outside your GMF-U**?

Maximum duration **in days**

#### What is the maximum duration of this absence during a training period outside your GMF- U

Maximum duration **in days**


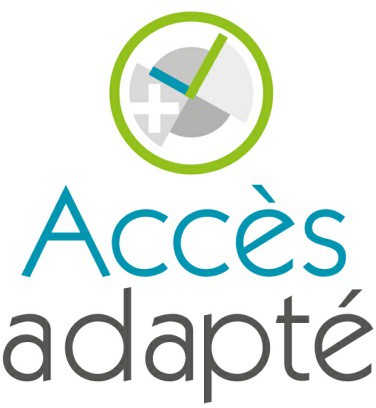


# Access adapted for GMF-U residents.

## Residence model in your GMF-U - continued

#### How is the **replacement of residents** during extended periods of absence (e.g. vacations, hospital internships, externships, illnesses) planned in the following situations:

*Tick the answer that applies most often*

Replacement ReplacementReplacement Reference

For routine patient follow-up

Responding to urgent patient needs

For lab/imaging readouts requiring rapid response

Replacement by a designated resident

by a designated group of residents

by any available resident

Replacement by supervisor

by another professional (e.g. IPS)

without appointment


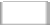

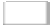

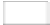

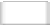

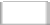

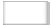

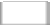

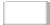

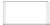

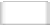

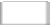

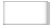

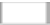

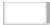

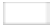

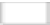

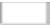

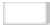


Description of residents' offices

How many minutes are allocated to residents in the following contexts?

In R1 In R2


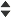

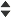


**Number of minutes allocated** to patient follow-up in a half-day's office work

**Number of minutes allocated** for initial assessment of a new patient


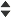

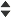


**Number of minutes allocated** for a regular follow-up appointment

**Number of minutes allocated** to a walk-in assessment


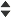

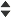

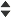

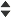


#### On the eve of a typical general family medicine clinic day, how much of the resident's schedule is left free in anticipation of patients' urgent or semi-urgent needs?


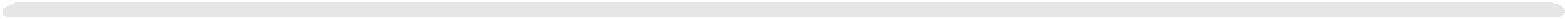

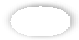

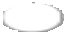


0%

50%

100%

In general, over a window of **how many weeks** is **a** resident's schedule open for appointments?

Number of weeks

Description of patient base

Are residents in charge of a **deﬁned group of patients**?


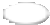
 Yes
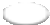
 No


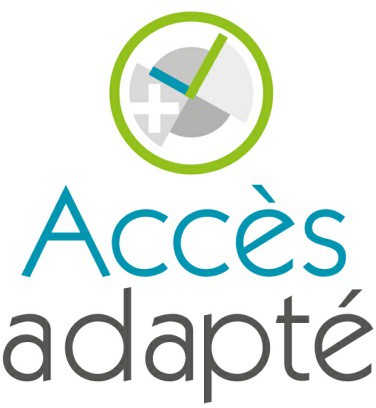


# Access adapted for GMF-U residents.

#### On average, **how many** patients are assigned to each resident's name?

Number of patients in R1:

Number of patients in R2:


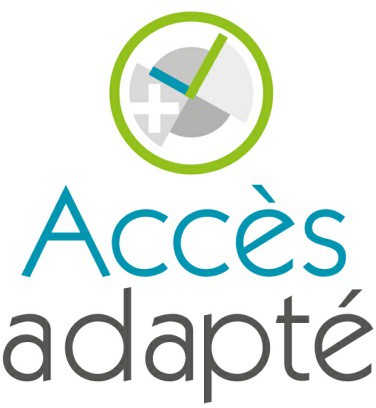


**Access adapted for GMF-U residents.**

In a typical week, what percentage of time slots is dedicated to these patients?


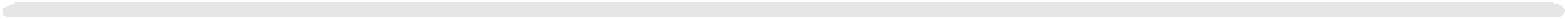

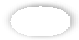

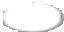


0%

50%

100%

Is there a formal patient allocation mechanism for residents?


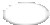
 Yes
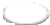
 No


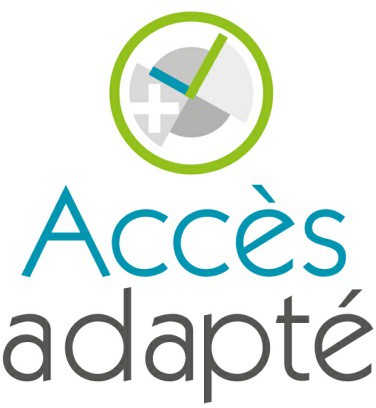


# Access adapted for GMF-U residents.

#### From the following options, choose the most appropriate allocation mechanisms.

*Check all that apply*


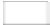
 Equitable distribution of patients according to classiﬁcation/cotation (e.g. vulnerability, pediatrics, geriatrics, pregnancy, etc.).


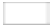
 Patient allocation based on acute care episode / walk-in visit
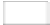
 Random patient allocation at start of residency


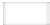
 Assigning a designated patient from a former resident


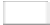
 Assigning patients continuously during residency
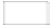
 Assigning patients to a group of residents


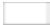
 Twinning with a supervisor's patient base
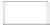
 Other (please specify)

Is the balance of the patient base (in terms of number or complexity) reviewed during the residency?


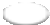
 Yes
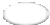
 No


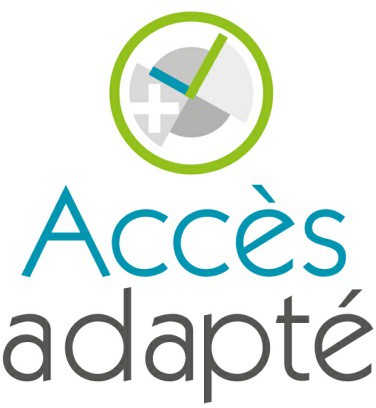


# Access adapted for GMF-U residents.

## Description of interprofessional collaboration

#### Is it possible to do joint follow-up with a professional other than a physician (e.g. mental health follow-up or follow-up of chronic conditions)?


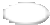
 Yes, right from the start in R1


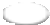
 Yes, but only at the ﬁn of R1
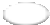
 Yes, from the beginning in R2


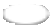
 Yes, but only in the ﬁn of R2


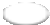
 Yes, this is possible depending on the nature of the pathology
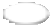
 No


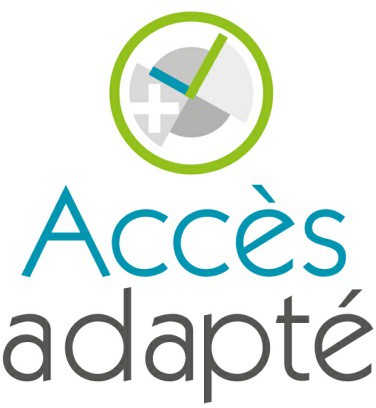


**Access adapted for GMF-U residents.**

Is it possible for residents in your GMF-U to oﬀrir joint follow-up with the following professionals?


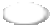

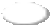

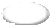
Yes No Not present at GMF-U


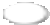

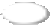

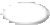


Inﬁrmière praticienne

Clinical nurse


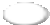

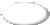

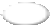


Auxiliary inﬁrmière

Social worker
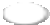

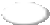

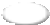


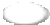

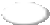

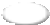


Pharmacist

Nutritionist
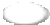

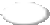

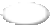


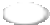

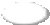

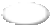


Physiotherapist

Occupational therapist
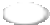

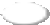

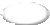


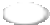


Psychologist

Sexologist

Respiratory therapist

Other specialists

Other professionals

Other (please specify type of professional)

**Access adapted for GMF-U residents.**

Do you have the option of using group prescriptions (e.g. anti-HTA, HGO medication adjustment by a clinical inﬁrmière)?

No

Yes, right from the start in R1

Yes, but only at the ﬁn of R1 Yes, from the beginning in R2

Yes, but only in the ﬁn of R2

Yes, this is possible depending on the nature of the pathology.

**Teaching adapted access in your GMF-U**

#### Is adapted access taught in your GMF-U?

Yes No

# Access adapted for GMF-U residents.

**How many hours are** devoted to this teaching during the residency?

- Less than 2 hours
- Between 2 and 4 hours
- More than 5 hours
- Don't know

Is this training compulsory?

Yes No

# Access adapted for GMF-U residents.

#### In your opinion, does your residential model allow for the integration of adapted access concepts?

To see the adapted access model, [*click*](https://admin-prod.crcsis.ca/files/2020/03/ModeleAA_piliers.pdf) *here*

#### Use the box below for any comments or other information you'd like to share about resident-friendly access.
